# Reexpression of Let-7g MicroRNA Inhibits the Proliferation and Migration via K-Ras/HMGA2/Snail Axis in Hepatocellular Carcinoma

**DOI:** 10.1155/2014/742417

**Published:** 2014-03-04

**Authors:** Ke-ji Chen, Ying Hou, Kui Wang, Jun Li, Yong Xia, Xiao-yu Yang, Gang Lv, Xiang-Lei Xing, Feng Shen

**Affiliations:** Department of Hepatic Surgery, Eastern Hepatobiliary Hospital, Second Military Medical University, 225 Changhai Road, Shanghai 200438, China

## Abstract

Let-7 family microRNAs have been reported to be downregulated in human hepatocellular carcinoma in comparison with normal hepatic tissues. Among them, let-7g was identified as the lowest expression using real-time RT-PCR. However, the mechanism by which let-7g works in hepatocellular carcinoma remains unknown. Here, in our present study, we have had let-7g reexpressed *in vitro* in hepatocellular carcinoma cell lines MHCC97-H and HCCLM3 via transfection. The proliferation after reexpression of let-7g was assayed using MTT method; the migration and invasion after restoration were detected by wound-healing and Transwell assay, respectively. We found using Western-blotting that let-7g can regulate epithelial-mesenchymal transition (EMT) by downregulating K-Ras and HMGA2A after reexpresssion. Xenografted nude mice were used to observe whether or not reexpression of let-7g could have potential therapeutic ability. *In vivo*, to observe the association with let-7g expression and overall prognosis, 40 paired cases of hepatocellular carcinoma were analyzed using in situ hybridization (ISH). It was found that reexpression of let-7g can inhibit the proliferation, migration, and invasion significantly, and that low expression of let-7g was significantly associated with poorer overall survival. Taken together, let-7g could be used as a promising therapeutic agent *in vivo* in the treatment of hepatocellular carcinoma at the earlier stage.

## 1. Introduction

Hepatocellular carcinoma (HCC) is the sixth most common and most aggressive malignancy and is the third leading cause of cancer-related deaths worldwide [1], with 5-year survival being poor. The tumorigenesis of HCC is a multistage process where noncoding genes, particular microRNAs (miRNAs), and protein-coding genes were found to be deregulated in the development [2]. It has been reported that aberrant expression of miRNAs may also contribute to the development and progression of HCC [3]. Growing reports suggest that miRNAs may function as oncogenes whose expressions usually were found to be upregulated in HCC tissues [4, 5] or as tumor suppressor genes whose expressions were discovered to be downregulated in cancer tissues as compared with normal tissues in the development and progression of HCC [6, 7].

Let-7 family has been reported to be downregulated significantly in HCC [8] whose 9 members have been found in humans [9]. Among the 9 members of let-7 family, let-7g was reported to be significantly associated with metastasis of HCC and breast cancer [10, 11]. Despite the let-7g being reported to negatively regulate Bcl-xL expression and induce apoptosis in cooperation with anticancer drug targeting Mcl-1 in HCC [12] and to inhibit the cell migration in HCC through targeting collagen type I *α*2 [10], the underlying mechanism by which let-7g works to inhibit proliferation and migration in HCC remains largely unknown.

In the present study, we have identified that expression of let-7g was lowest among the seven let-7 family members that we have chosen in terms of basal expression in clinical HCC tissues using the real-time RT-PCR, and that it is only let-7g that is significantly associated with metastasis of HCC. We found that reexpression of let-7g could alleviate the epithelial-mesenchymal transition (EMT) via downregulating the K-Ras/HMGA2A pathway. In order to evaluate the potential therapeutic ability of let-7g, xenografted nude mice were employed. It was found that let-7g can significantly suppress the tumorigenesis of HCC *in vitro* at the earlier stage. Furthermore, we found that low expression of let-7g was significantly associated with poorer survival outcomes.

## 2. Materials and Methods

### 2.1. HCC Cell Lines and Clinical Tissues Samples

The human HCC cell lines MHCC97-H and HCCLM3 (Shanghai fmgbio, Shanghai, China) were cultured in Dulbecco's modified Eagle's medium (DMEM, Invitorgen, Carlsbad, CA) containing 10% fetal bovine serum (FBS) in a humidified incubator at 37 with an atmosphere of 5% CO_2_. 40 pairs of fresh samples from HCC were recruited from surgical specimens collected from 2007 to 2013, fixed in formalin, and embedded in paraffin. The current study was approved by the local Medical Ethics Committee and signed informed consent was obtained. None of the recruited patients received treatment before surgery, and for all patients clinical-pathological information was available.

### 2.2. RNA Isolation and qRT-PCR

Total RNA of cells was isolated using Trizol reagent (Invitrogen, CA, USA) according to the manufacturer's instructions. RNA was reversely transcripted into cDNAs by PrimeScript one-step RT-PCR kit (TAKARA, Dalian, China). The expression of let-7g was measured by qRT-PCR assays. All samples with SYBR Green PCR Master Mix (TAKARA, Dalian, China), U6 expression was assayed for normalization, and relative gene expression determinations were made with the comparative delta-delta CT method (2^−ΔΔCt^). The reaction mixtures of let-7g and U6 were incubated at thermal cycling conditions comprised 95°C for 30 sec, and 40 cycles at 95°C for 5 sec followed by 56.5°C for 30 sec. The primers used for quantitative RT-PCR were provided in Supplementary Table  1 (available online at http://dx.doi.org/10.1155/2014/742417).

### 2.3. In Situ Hybridization (ISH)

Expression of let-7g in 40 paired of HCC tissues and normal controls were detected by ISH with probes for let-7g (Exiqon, Woburn, Massachusetts). Melted paraffin was in an oven at 60°C for 45 min then stored as slides overnight (O/N) at 4°C. Deparaffinized slides were in xylene and ethanol solutions at room temperature and then incubated with Proteinase-K for 10 min at 37°C. Slides were hybridized with 20 nmol/L let-7g probe in a hybridization buffer for 2 h at 55°C then washed with SSC buffers. The remaining procedures were performed with a modified version of the manufacturer's protocol. The slides were counterstained with hematoxylin and eosin (H&E) and visualized under a microscope. Each slide was examined by an observer blinded to the diagnosis and clinicopathologic data and reviewed and confirmed by a second blinded observer. Staining intensity and percentage of positive cells were noted as follows: four grades were used for the staining intensity (0: no intensity; 1: weak intensity; 2: moderate intensity; 3: strong intensity), and four grades were used for the percentage of positive cells (0: less than 10%; 1: between 10%–25%; 2: between 25%–40%; 3: more than 40%). HCC patients were classified into two groups according to the total score of staining intensity plus percentage of positive cells: low expression group (total score: 0–2) and high expression group (total score: 3–6), in order to better analyze the prognosis between groups.

### 2.4. Transfection

Plasmid vector pCMV-let-7g harboring let-7g precursor and scramble sequence were purchased from OriGene (SC400010, OriGene, USA). MHCC97-H and HCCLM3 cells were grown to 80–90% confluence in 6-well plates and were transfected with 4.0 *μ*g of pCMV-let-7g or scramble control vector together with 10 *μ*L Lipofectamine 2000 (Invitrogen, CA, USA) in Opti-MEM (Invitrogen, CA, USA) following the manufacturer's instruction (Invitrogen Life Technologies, CA, USA). All groups were performed in triplicate. Cells were lysed for RNA at 48 h and protein at 72 h after transfection.

### 2.5. Western Blotting

Seventy-two hours after transfection, MHCC97-H and HCCLM3 cells were harvested in RIPA Lysis buffer (Bioteke, Beijing, China) and 80 *μ*g of cellular protein was subjected to 10% SDS-PAGE separation. Proteins were transferred to PVDF microporous membrane (Millipore, Boston, MA, USA) and blots were probed with rabbit polyclonal antibody against K-Ras (#3339), ERK1/2 (#4695), p-ERK1/2 (#4370), Snail (#4719), E-cadherin (#3195), N-cadherin (#4061), and HMGA2 (#8179); they were all from cell signaling technology (Cell Signaling Technology, USA). *β*-Tubulin (sc-9104), GAPDH (sc-25778), Bax (sc-20067), Bak (sc-1035), and Bcl-xL (sc-8392) were all from Santa Cruz Biotechnology (CA, USA). *β*-Tubulin and GAPDH were chosen as an internal control. the blots were visualized using WesternBreeze Kit (WB7105, Invitrogen Life Technologies, CA, USA), and further quantified using Quantity One Software (Bio-Rad Laboratories, USA) by measuring the band intensity for each group and normalizing to *β*-Tubulin and GAPDH as internal control. The final results were expressed as fold changes by normalizing the data to the control values.

### 2.6. Cell Proliferation Assay

The methyl thiazolyl blue tetrazolium (MTT; Sigma-Aldrich, St Louis, MO) spectrophotometric dye assay was used to detect cell proliferation ability. MHCC97-H and HCCLM3 cells were plated in 96-well plates at a density of 5 × 10^3^ cells per well. After transfection experiments, cell proliferation was assessed. Cells were incubated for 4 h in 20 *μ*L MTT at 37°C. The color was developed by incubating the cells in 150 *μ*L dimethyl sulfoxide, the absorbance was detected at 490 nm wave length. The data were obtained from three independent experiments.

### 2.7. Cell Migration and Invasion Assays *In Vitro*

Cell migration was assayed using the wound healing assay. MHCC97-H and HCCLM3 cells were plated in 6-well plate at a concentration of 5 × 10^5^ cells/well and allowed to form a confluent monolayer for 24 h. After transfection, the monolayer was scratched with a sterile pipette tip (10 *μ*L) then washed with serum free medium to remove the floating and detached cells and photographed (time 0 h, 24 h and 48 h) using inversion fluorescence microscope (Olympus, Japan). Cell culture inserts (24-well, pore size 8 *μ*m; BD Biosciences) were seeded with 5 × 10^3^ cells in 100 *μ*L of medium with 0.1% FBS. Inserts precoated with Matrigel (40 *μ*L, 1 mg/mL; BD Biosciences) were used for invasion assays. Medium with 10% FBS (400 *μ*L) was added to the lower chamber and served as a chemotactic agent. Noninvasive cells were wiped from the upper side of the membrane and cells on the lower side were fixed in cold methanol (−20°C) and air dried. Cell were stained with 0.1% crystal violet (dissolved in methanol) and counted using the inverted microscope. Each individual experiment had triplicate inserts, and 4 microscopic fields were counted per insert.

### 2.8. Cell Cycle and Apoptosis Analysis

The cell cycle and apoptosis were analyzed using flow cytometry (FCM). For cell cycle analysis, cells were plated at a density of 3 × 10^5^ per well in 6-well plate and transfected with either PCMV-let-7g overexpression plasmid or a blank vector control. Cells were washed twice with cold Phosphate buffer saline (PBS) and fixedin 70% cold ethanol O/N at 4°C. After incubation with RNase for 1 h at 4°C, DNA was stained with 2 *μ*L propidium iodide (PI) (400 mg/mL) for 15 min then was analyzed by FCM. For cell cycle analysis, Annexin V-FITC apoptosis detection kit was used (Invitrogen Life Technologies, CA, USA), and AnnexinV-FITC staining was performed following the instructions provided by the manufacturer. Briefly, cells were washed twice with cold PBS and resuspended in 400 *μ*L with 1× binding buffer at a concentration of 1 × 10^6^ cells/mL. Cells were then mixed with 5 *μ*L of the Annexin V-FITC solution and 2 *μ*L of propidium iodide (PI). Cells were incubated for 15 min at 4°C in the dark then analyzed by FCM.

### 2.9. Tumor Xenografts

To evaluate *in vivo* tumorigenesis, HCC xenografting mouse model was developed. Male BALB/c-nude mice of 4 weeks were prepared for tumor implantation. All animals were maintained in a sterile environment on a daily 12-h light/12-h dark cycle and categorized into 2 groups with each group being 8 nude mice. After resuspension in PBS, HCCLM3 cells (3 × 10^6^/mouse) were injected subcutaneously into the flanks of the nude mice. One week after implantation when the tumor became visually palpable at the size of 2 mm in diameter, intratumoral injection with 40 *μ*g of let-7g precursor plasmid dissolved in 100 *μ*L of DMEM mixed with 5 *μ*L of Lipofectamine 2000 (Invitrogen) was done every two days. Tumor volume (TV) was measured every two days according to the formula: TV (mm^3^) = length × width^2^ × 0.5 [13].

### 2.10. Statistics

Data were expressed as mean ± SD and were analyzed by Student's *t*-test, one-way ANOVA, and *χ* ^2^ test using SPSS for Windows version 16.0 (SPSS, Chicago, USA). Kaplan-Meier survival curves were plotted and log rank test was done. The significance of various variables for survival was analyzed by Cox proportional hazards model in a multivariate analysis. *P* < 0.05 in all cases was considered statistically significant.

## 3. Results

### 3.1. Let-7 Family Were Reduced in HCC

**Figure 1:**
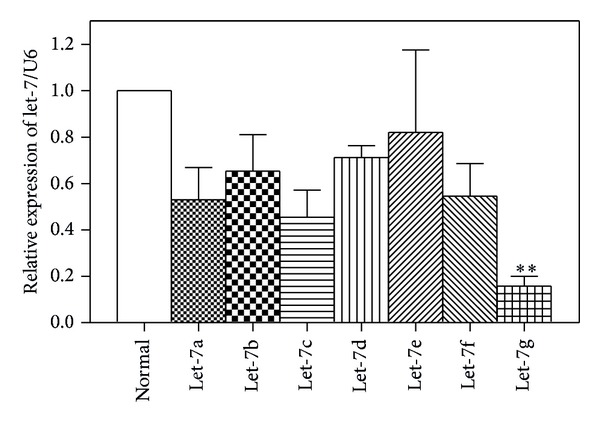
The endogenous expression level of let-7g in HCC tissues. The basal expression of let-7 family members, including let-7a, let-7b, let-7c, let-7d, let-7e, and let-7f was detected using quantitative real-time Reverse Transcription PCR (qRT-PCR) in 40 paired HCC tissues and their normal controls. Total RNA was extracted using Trizol reagent after 48 h. The relative expression of let-7 family members, normalized to U6, was calculated using the formula 2^−ΔΔCt^ (relative expression). **P < 0.01 versus normal control.

To observe the basal expression of let-7 family in HCC, we performed real-time RT-PCR to detect the 7 members of let-7 miRNA family in 40 paired HCC clinical tissues and paired normal control. It was found that the levels of miRNA let-7 family including let-7a, let-7b, let-7c, let-7e, let-7f, and let-7g were all consistently reduced in HCC tissues as compared with the normal hepatocellular tissues. Among them, relative expression of let-7g was significantly the lowest (Figure 1). Hence, let-7g was chosen as miRNA of interest in our following experiment.

### 3.2. Low Expression of Let-7g Was Significantly Associated with Prognosis

**Figure 2:**
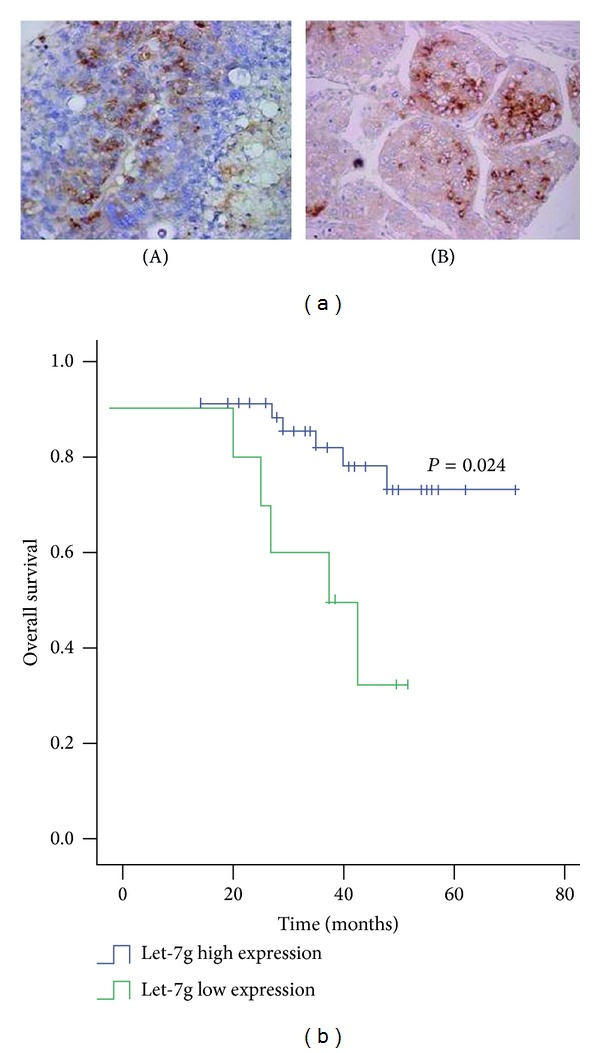
Low expression of let-7g was significantly associated with poorer overall survival. (a) Heterogeneous expression of let-7g was detected using in situ hybridization method in HCC tissues. Figure (A) showed that let-7g was low in HCC whereas figure (B) showed let-7g high in HCC. The magnification fold was (×100). (b) Kaplan-Meier survival curves were plotted. There is strikingly significant difference between let-7g positive group and let-7g negative group (*P* < 0.01, using log rank test), after analysis using ISH in 40 cases of HCC tissues.

To investigate the expression and localization of let-7g in patients with HCC, in situ hybridization (ISH) was performed. It can be seen that let-7g was mainly localized in cytoplasm of HCC cells and let-7g was heterogeneously expressed, with some cases being high and others being low expression in HCC tissues (Figure 2(a)). Kaplan-Meier analysis was conducted illustrating that there was significant correlation between poor overall survival and low expression of let-7g in the 40 cases of HCC cohort using ISH method (Figure 2(b)).

### 3.3. Reexpression of Let-7g Inhibited Proliferation in HCC Cell Lines

**Figure 3:**
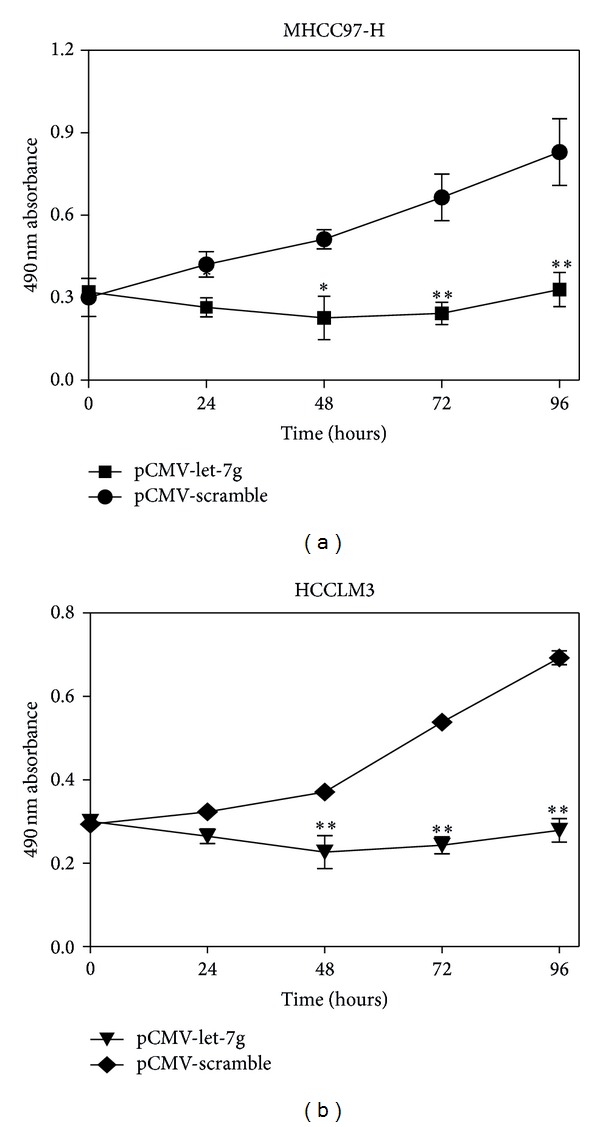
Reexpression of let-7g inhibited the proliferation of MHCC97-H and HCCLM3 cells. Cell proliferation of MHCC97-H and HCCLM3 cells after reexpression of let-7g for 0 h, 24 h, 48 h, 72 h, and 96 h were examined by MTT assay. Let-7g can obviously retard the proliferation as compared with control group (**P < 0.01, one-way ANOVA analysis). The mean and standard error from triplicate experiments are indicated.

To elucidate the functional role of let-7g in HCC, eukaryotic expression vector harboring let-7g precursor sequence and control vector harboring scramble sequence of let-7g were transfected into two different kinds of HCC cell lines, MHCC97-H and HCCLM3, respectively. Both the two vectors had green fluorescent protein (GFP), which can be indirectly monitoring the expression rate after transfection (Supplementary Figure  1). Efficiency of transfection was measured using quantitative real-time RT-PCR (Supplementary Figure  2). The cell proliferation was monitored using MTT method continuously for 4 days after transfection. No significant changes in size and morphology of MHCC97-H and HCCLM3 were found using light microscope (data not shown). However, the proliferation in the group transfected with let-7g precursor plasmid was significantly suppressed as compared with the group transfected with scramble sequence of let-7g for MHCC97-H and HCCLM3 cell lines (Figure 3).

### 3.4. Reexpression of Let-7g Inhibited Migration and Invasion of HCC Cell Lines

**Figure 4:**
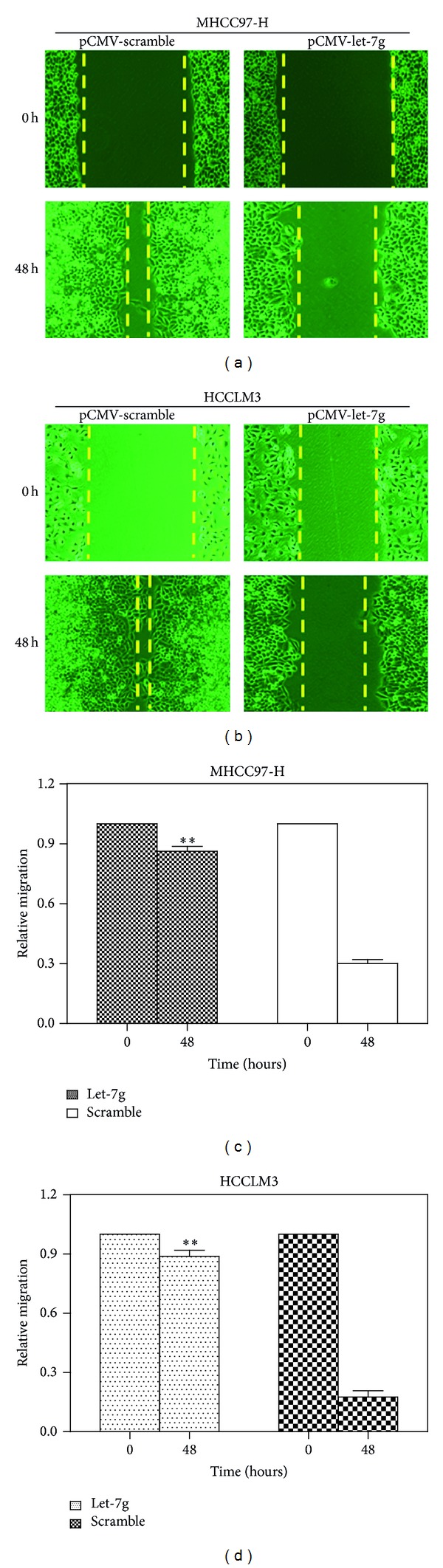
Reexpression of let-7g inhibited the migration of MHCC97-H and HCCLM3 cells. (a) Qualification of wound-healing assay of MHCC97-H after re-expression of let-7g for 0 and 48 hours. (b) Qualification of wound-healing assay of HCCLM-3 after re-expression of let-7g for 0 and 48 hours. (c) Quantitation of wound-healing assay of MHCC97-H. Migration of MHCC97-H cells was significantly retarded in comparison with scramble sequence control group. There is strikingly significant difference between group transfected with scramble sequence and group transfected with let-7g group (**P < 0.01, one-way ANOVA analysis). (d) Quantitation of wound-healing assay of HCCLM-3. Highly similarly, migration of HCCLM-3 cells was also significantly suppressed compared with scramble sequence group. (**P < 0.01, one-way ANOVA analysis). The mean and standard error from triplicate experiments are indicated.

**Figure 5:**
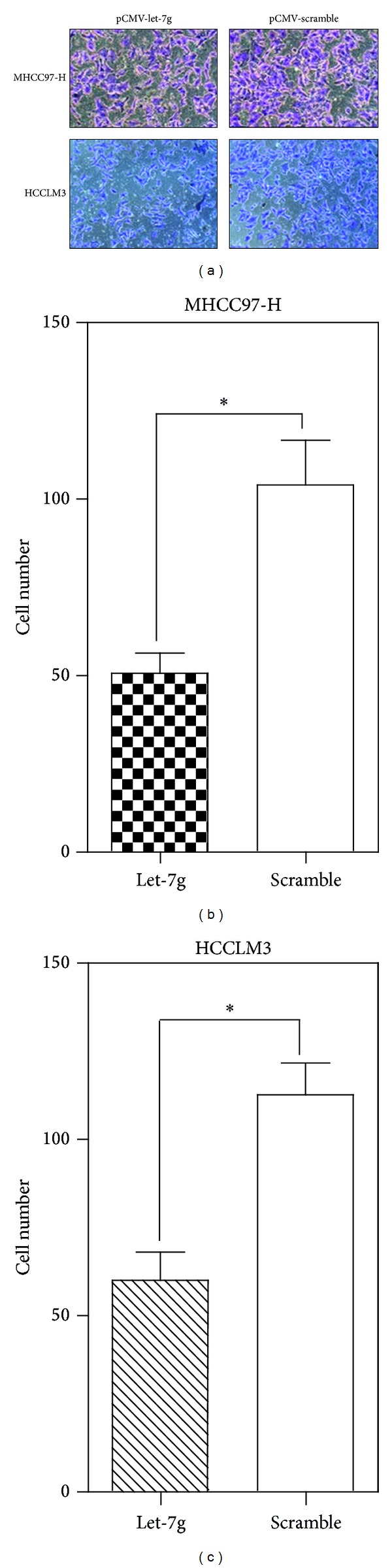
Reexpression of let-7g inhibited the invasion of MHCC97-H and HCCLM3 cells. (a) Qualification of Transwell assays for MHCC97-H and HCCLM3 after reexpression of let-7g for 48 hour. (b) Quantitation of Transwell assay for MHCC97-H (*P < 0.05, Student's *t*-test). (c) Quantitation of Transwell assay for HCCLM3 (*P < 0.05, Student's *t*-test). The mean and standard error from triplicate experiments are indicated.

To test whether or not let-7g reexpression could have an effect on the metastasis of HCC cell lines, we examined the rate of migration and invasion through wound-healing and Transwell approach. It was found that the migration (*P* < 0.01, Figure 4) and invasion (*P* < 0.05, Figure 5) were both significantly inhibited in MHCC97-H and HCCLM3 cell lines after reexpression of let-7g.

### 3.5. Reexpression of Let-7g Induced Apoptosis and Cell Cycle Change of HCC Cell Lines

**Figure 6:**
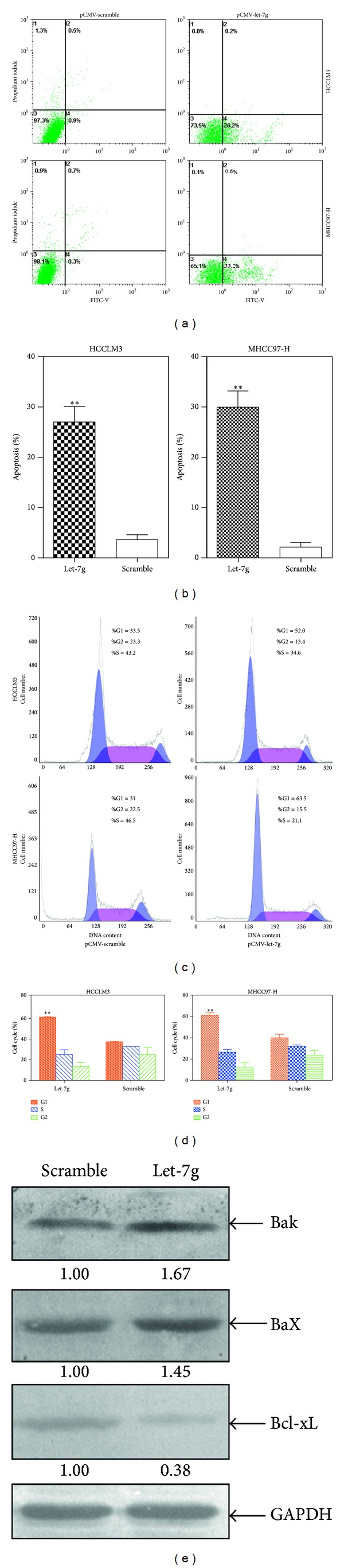
Reexpression of let-7g induced cell apoptosis and changes of cell cycle. (a) Qualification assay of cell apoptosis of MHCC97-H and HCCLM3 after reexpression of let-7g. (b) Quantitation assay of cell apoptosis of MHCC97-H and HCCLM3 after re-expression of let-7g (**P < 0.01, Student's *t*-test). (c) Qualification assay of cell cycle of MHCC97-H and HCCLM3 after reexpression of let-7g. (d) Quantitation assay of cell cycle of MHCC97-H and HCCLM3 after reexpression of let-7g (**P < 0.01, Student's *t*-test). (e) Biochemical analysis of proteins that are reported to be involved in the regulation of cell cycle. The representative results out of three times experiments were shown here. 80 *μ*g total protein was loaded per lane when performing Western-blotting.

To examine whether or not reexpression of let-7g could induce cell apoptosis and cell cycle variation, both MHCC97-H and HCCLM3 cell lines were subjected to Flow Cytometry analysis after reexpression of let-7g for 48 hours. We found that significant apoptosis occurred to both of the two different HCC cell lines (*P* < 0.01, Figures 6(a) and 6(b)). With regard to variation of the cell cycle, it was found that cell cycles were arrested at G1 stage (*P* < 0.01, Figures 6(c) and 6(d)). Based on the earlier reports [12], several molecular markers involved in the regulation of apoptosis and cell cycle were detected using Western-blotting. What was previously reported was confirmed; that is, apoptosis related proteins BaX and BaK were upregulated, whereas apoptosis inhibiting protein Bcl-xL was downregulated after reexpression of let-7g precursor for 72 hours (Figure 6(e)), suggesting that it is through upregulating the Bax and BaK and downregulating Bcl-xL that let-7g induced apoptosis of HCC cell lines, MHCC97-H and HCCLM3.

### 3.6. Reexpression of Let-7g Suppressed the Expression of HMGA2 and EMT Markers

**Figure 7:**
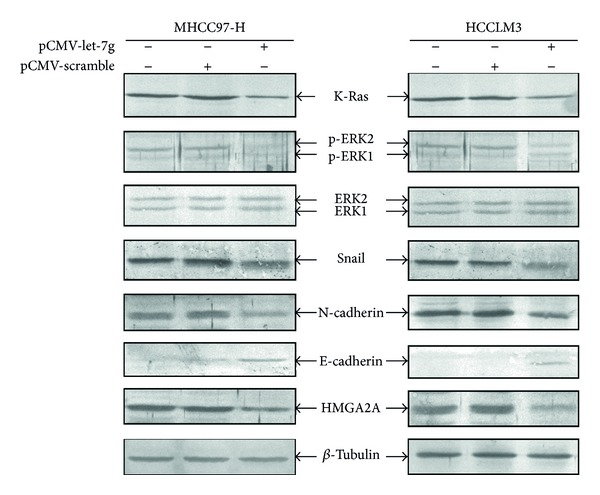
Reexpression of let-7g regulated EMT through downregulating the K-Ras/ERK1/2 signaling pathway. Western-blot analysis of K-Ras, p-ERK1/2, ERK1/2, Snail, E-cadherin, N-cadherin, and HMGA2A after transfection with let-7g precursor and scramble sequence control plasmids for 72 hours. *β*-Tubulin was as loading control. 80 *μ*g total protein was loaded per lane, which was separated by 10% SDS-PAGE followed by visualization with WesternBreeze kit (Invitrogen, USA).

To explore the possible mechanism of let-7g, we hypothesized that let-7g reexpression inhibited malignant cellular behaviors via downregulating K-Ras-mediated mitogen-activated protein kinase (MAPK) signaling pathway, based on the analysis and reanalysis of published reports available [14–16]. Additionally, a recent study has shown that HMGA2, one of the targets of let-7g, was involved in the process of epithelial-mesenchymal transition (EMT) [17]. In the light of these peer findings mentioned; we detected some of the key protein markers which were reported to be associated highly with MAPK and EMT using immunoblotting approach. it was shown that K-Ras and phosphorylated ERK1/2 (p-ERK1/2) were decreased in both MHCC97-H and HCCLM3 cell lines after reexpression of let-7g as compared with control group. On reexpressing the let-7g, E-cadherin that has been a typical pathological marker for epithelial trait was upregulated; meanwhile, N-cadherin and Snail were downregulated. HMGA2A expression was expectedly and significantly decreased after upregulation of let-7g (Figure 7), which was wholly consistent with Zucchini-Pascal et al.'s observation [18].

### 3.7. Reexpression of Let-7g Suppressed Tumorigenesis of HCC Xenografts

**Figure 8:**
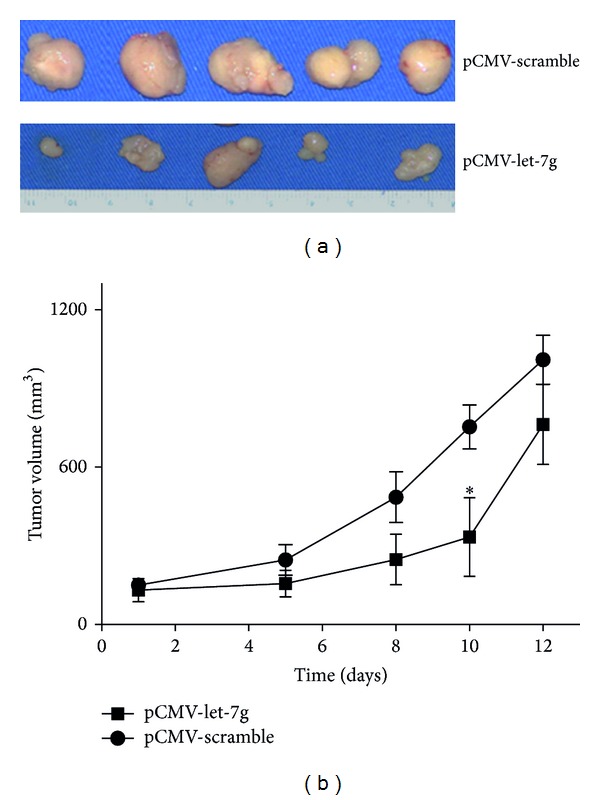
Reexpression of let-7g inhibited the tumorigenesis of HCC in xenografted nude mice. Every 2 days intratumoral injection with let-7g precursor plasmid significantly reduced the tumor volume at the 10th day as compared with control plasmid group. (a) Representative image of tumors resected from the two groups. (b) Quantitative evaluation of tumor size at different therapeutic times (*P < 0.05, Student's *t*-test).

Next, to test whether or not let-7g re-expression could suppress the tumor formation *in vivo*, nude mice xenografted with HCCLM3 cells were employed. Intratumoral injection with let-7g precursor plasmid at two-day interval was as experimental group. Injection with plasmid having scramble sequence as in the same way control. Tumor size was calculated according to the reference [13]. It can be seen that injection with let-7g precursor plasmid can significantly reduce the tumor volume of HCCLM3 before or at the 10th day (Figure 8), suggesting that reexpression of let-7g could be used as ideal therapeutic agent in the treatment of HCC at the earlier stage.

## 4. Discussion

In our present study, we found that among the let-7 family miRNAs, endogenous expression of let-7g was lowest in the clinical HCC tissues. Reexpression of let-7g can significantly inhibit the malignant behaviors of HCC cells *in vitro* and suppress HCC tumorigenesis at the earlier stage *in vivo*. Further, in HCC cell lines, we found it is the way through K-Ras/HMGA2A/Snail axis that reexpression of let-7g inhibitied the proliferation, migration, and invasion of HCC cells. Additionally, low expression of let-7g was significantly associated with inferior overall survival of patients with HCC. Our results suggest that reexpression of let-7g could be used as an ideal therapeutic agent in the management of HCC at earlier stage.

Let-7 is a family consisting of 13 members located on nine different chromosomes whose expression usually has been lost, reduced, or deregulated in the majority of human cancers [2]. A growing evidence suggests that restoration of let-7 expression has an antiproliferative effect on cancer cells of different kinds [8], thus indicating that let-7 restoration may be a useful therapeutic option in HCC. In light of the conflicting expression patterns of let-7 family across human cancers [8], we first tested the expression levels of seven kinds of let-7 family members we have chosen in our study in 40 paired HCC tissues and their corresponding normal controls. We observed that the 7 different members of let-7 family were all reduced in HCC tissues as compared with normal control tissues. Among these, we have identified that only let-7g was significantly correlated with HCC metastasis, which was highly consistent with result obtained by Zhao and colleagues that let-7g was found to be reduced and associated with metastasis of HCC [19]. We therefore hypothesized that the low let-7g may contribute to the high metastasis rate of HCC cells.

To test the hypothesis we proposed, we transfected the two different HCC cell lines, MHCC97-H and HCCLM3 with the similar metastatic ability, with let-7g precursor plasmid and control plasmid, respectively. It is shown that reexpression of let-7g in MHCC97-H and HCCLM3 can significantly suppress the migration and invasion ability of MHCC97-H and HCCLM3 cells in culture system, which was in line with and in agreement with Qian and coworkers' report in spite of whose consistent results were found in breast carcinoma [11].

In the following, we have analyzed whether or not there were variations of cellular apoptosis and cell cycle after restoration of let-7g. We found that reexpression of let-7g can substantially induce apoptosis rate as compared with control group where scramble sequence of let-7g was transfected, and cell cycles were significantly arrested at G1 stage. Based on the earlier report [12] that let-7g could induce cellular apoptosis via downregulation of Bcl-xL and meanwhile upregulation of Bax. To verify the proposal, we have tested the protein markers mentioned previously in HCCLM3 after reexpression of let-7g. It turns out to be that our results confirmed the previous findings.

What is the potential mechanism by which reexpression of let-7g inhibited migration and invasion in HCC cell lines? So far, the potential mechanism by which let-7g work in the antiproliferation and antimetastasis of HCC still remains unknown, despite the recent peer findings available that let-7g may suppress HCC metastasis partially through targeting COL1A2 [10] and that let-7g inhibit cell proliferation of HCC by downregulating the c-Myc and upregulating p16 (INK4A) [20]. Thus, to better understand the potential mechanism of let-7g, we explored the variations of the several possible important protein markers that were reported to be involved in EMT process based on the evidence available [21, 22]. We found that EMT happened to both MHCC97-H and HCCLM3 cells. The epithelial trait marker, E-cadherin, was upregulated whereas at the same time N-cadherin and Snail which belonged to specific mesenchymal biomarker were correspondingly decreased in a different level, suggesting that restoration of let-7g could alleviate the EMT extent that is crucial event in HCC progression and recurrence [22]. Liu et al., in the investigation of EMT of breast cancer, discovered the Ras was heavily involved in EMT process [23]. Based on reports above mentioned, we found that E-cadherin, N-cadherin, and Snail were changed as reported after K-Ras was transiently silenced using specific siRNA. Tan and colleagues made serial discoveries that HMGA2 can regulate important transcription factor Twist [24] and Snail [25] in a single or combinational with Smads in the induction of EMT. Therefore, based on findings of our own as well as other peer findings, results suggest that reexpression of let-7g could suppress the EMT process via downregulation of K-Ras/ERK1/2 signaling pathway.

Given the significant anti-proliferation effect of let-7g, we have developed the xenografted nude mice to evaluate the effect after reexpression of let-7g *in vivo* in the tumorigenesis of HCC. It was shown that let-7g reexpression can significantly inhibit the tumorigenesis of HCC in nude mice, which is highly consistent and similar with Kumar et al., in their study in the evaluation of suppression of tumorigenesis of let-7g in nonsmall cell lung cancer [14]. Nonetheless, one dilemma that is inevitable and we fell into in the experimentation of xenografted nude mice is that let-7g reexpression was only workable at the earlier stage (at or before the 10th days). After the 10th day, it turns out to be insufficient or incapable of inhibiting the tumorigenesis of HCC. Thus, it can be seen that the continuous let-7g delivery might lead to initial suppression of tumor growth before the 10th day but let-7g-resistant tumors would eventually and inevitably emerge. The active effect of let-7g may be related to the drug infiltration and microenvironment change of the tumors [26]. In spite of the limitation of the results, our study indicates that let-7g could still be used as an ideal therapeutic agent *in vivo* in the earlier stage therapy of HCC.

In our present study, we have employed 40 cases of HCC tissues and their controls, although quite limited, observing that low expression of let-7g was significantly associated with poorer overall survival. The result should have been required to be confirmed with a great larger number of patients with HCC in multidisciplinary centers.

Put together, we found that restoration of let-7g can significantly inhibit the malignant behaviors of HCC cells *in vitro* in the way through downregulating the K-Ras/HMGA2A/Snail axis and suppressing HCC tumorigenesis *in vivo *at the earlier stage. What is more is that low expression of let-7g was significantly associated with inferior overall survival of patients with HCC. Our results suggest that let-7g could be used as an ideal therapeutic agent *in vivo* in the management of patients with earlier stage of HCC.

## Supplementary Material

Primers used for quantitative real-time RT-PCR (supplementary table 1) and plasmids transfection rate and effect was determined by fluorescence reverse microscope (supplementary figure 1) and qRT-PCR (supplementary figure 2) respectively.
