# Supplementary material for: Reexpression of Let-7g MicroRNA Inhibits the Proliferation and Migration via K-Ras/HMGA2/Snail Axis in Hepatocellular Carcinoma
Source: Biomed Res Int. 2014 Mar 4;2014:742417. doi: 10.1155/2014/742417 (PMC3960551; doi:10.1155/2014/742417)

Supplementary

Table 1. Primers used for quantitative RT-PCR.

**Primers**	**Sequences**
U6 forward	ctcgcttcggcagcaca
U6 reverse	aactcttcactaatttgctg
let-7a	tgaggtagtaggttgtatagtt
let-7b	tgaggtagtaggttgtgtggtt
let-7c	tgaggtagtaggttgtatggtt
let-7d	agaggtagtaggttgcatagtt
let-7e	tgaggtaggaggttgtatagtt
let-7f	tgaggtagtagattgtatagtt
let-7g	tgaggtagtttgtacagtt

Figure 1

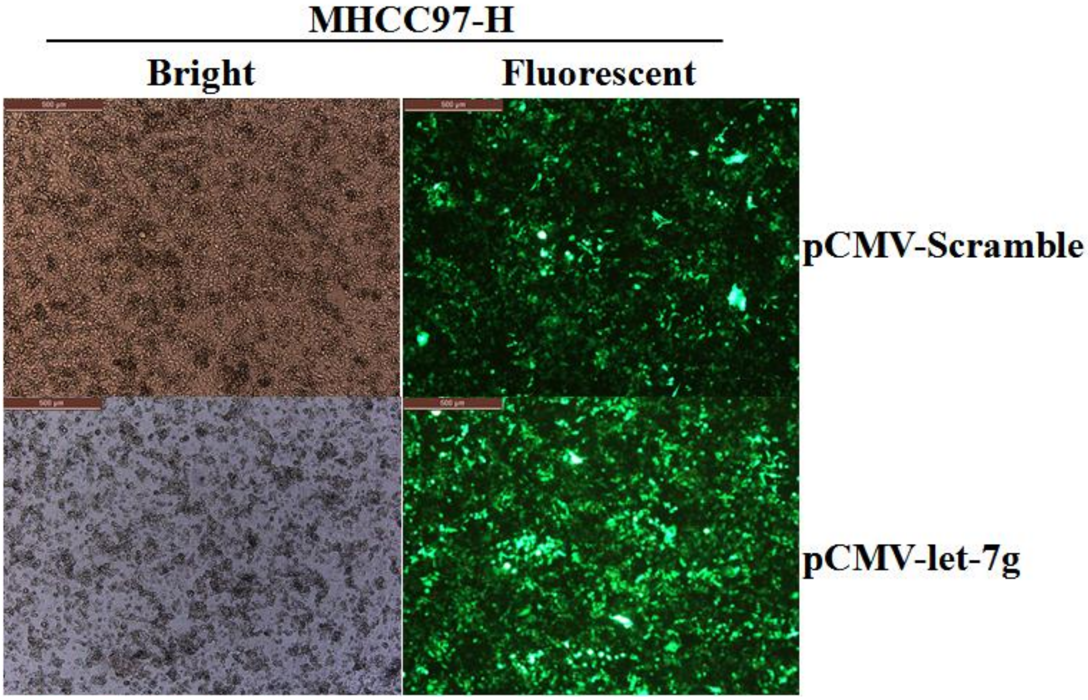

MHCC97-H
Bright
Fluorescent
500 μm
500 μm
500 μm
500 μm
pCMV-Scramble
pCMV-let-7g

Figure 2

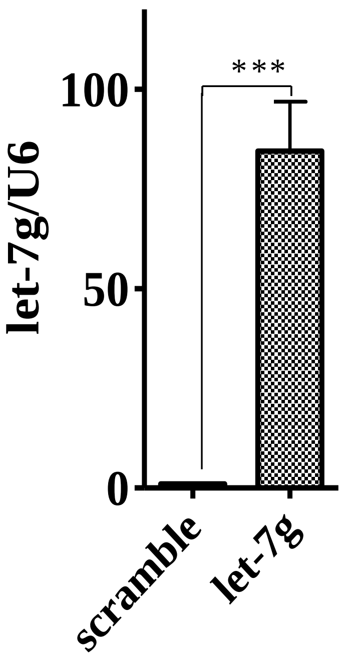

Supplement: Supplementary file 1 — Primers used for quantitative real-time RT-PCR (supplementary table 1) and plasmids transfection rate and effect was determined by fluorescence reverse microscope (supplementary figure 1) and qRT-PCR (supplementary figure 2) respectively. [file 742417.f1.pdf]
